# Interleukin-4 expression is increased in patients with tuberculosis: A systematic review and meta-analysis

**DOI:** 10.1097/MD.0000000000034041

**Published:** 2023-06-16

**Authors:** Jie He, Lingmeng Song, Pengcheng Zheng

**Affiliations:** a Clinical Medical College of Chengdu Medical College, Chengdu, China; b Department of Pulmonary and Critical Care Medicine, The First Affiliated Hospital of Chengdu Medical College, Chengdu, China; c Sichuan Clinical Research Center for Geriatrics, Chengdu, China; d Medical Department, The First Affiliated Hospital of Chengdu Medical College, Chengdu, China.

**Keywords:** active tuberculosis, interleukin-4, latent tuberculosis, meta-analysis, systematic review

## Abstract

Interleukin-4 (IL-4) is an important cytokine in the Th2 differentiation of CD4^+^ T cells, which modulates immune responses and participates in host defense against Mycobacterium tuberculosis. The present study aimed to evaluate the significance of IL-4 concentration in patients with tuberculosis. Data from this study will be helpful in understanding the immunological mechanisms of tuberculosis and in clinical practice.

**Method:**

A data search was conducted from January 1995 to October 2022 in electronic bibliographic databases such as China National Knowledge Infrastructure, Wan Fang, Embase, Web of Science, and PubMed. The Newcastle-Ottawa Scale was used to assess the quality of the included studies. Heterogeneity between the studies was assessed using *I*^2^ statistics. Publication bias was determined by funnel plot, and Egger’s test was used to confirm the presence of publication bias. All qualified studies and statistical analyses were performed using Stata 11.0.

**Results:**

Fifty-one eligible studies comprising 4317 subjects were included in the meta-analysis. The results depicted a considerably increased level of serum IL-4 in patients with tuberculosis than in the controls (standard mean difference [SMD] = 0.630, [95% confidence interval (CI), 0.162–1.092]). However, there was no significant difference in plasma IL-4 levels between patients with TB and controls (SMD = 0.290, [95% CI, −0.430 to 1.010]). In addition, the infection status, TB focus location, drug resistance, race, research design type, and detection method divided the subjects into different subgroups for the meta-analysis. The results of the comparison of healthy controls and TB subjects showed that in the Asian population, the serum IL-4 level in patients with TB was higher than that in controls (SMD = 0.887, [95% CI, 0.202 to −1.573]) and patients with active TB as well as people with pulmonary TB showed increased serum IL-4 levels compared to controls (SMD = 0.689, [95% CI, 0.152–1.226]). In the case of the control group with latent TB, the active TB group had higher serum IL-4 levels than the control group (SMD = 0.920, [95% CI, 0.387–1.452]).

**Conclusion:**

The present meta-analysis showed that serum IL-4 varied in healthy individuals and patients with TB. Patients with active TB may also exhibit higher IL-4 concentrations.

## 1. Introduction

Presenting as an infectious disease, tuberculosis (TB) is a common clinical chronic disease, with the cause being Mycobacterium tuberculosis (MTB) and a hidden onset.^[1]^ MTB kills 1.5 million people annually. An estimated 5% to 10% of the latently MTB-infected one-third of the global population develop active TB.^[2,3]^ In many cases, the lack of reliable monitoring indicators that are relatively simple and dynamic remains a significant challenge for TB control despite progress in the area of TB biomarkers.^[4]^ Presently, the potential candidates for examining the response to anti-TB treatment, the severity of illness, and determining the risk of TB infection are mostly host-derived and not commercially available.^[5]^

The incidence of TB is closely associated with the body’s immune status. The balance between Th1/Th2 cellular immune responses plays a major role in the immune regulation of TB.^[6]^ Interleukin-4 (IL-4) is a pleiotropic cytokine^[7]^ produced by activated T lymphocytes, mast cells, and basophils. It can promote and maintain the proliferation of Th2 cells, induce a humoral immune response, inhibits the immune response of Th1 cells to nodules, and impede the host’s ability to control bacterial numbers.^[8]^ All these interactions might eventually produce adverse effects on tuberculosis patients.^[9]^ Higher production of IL-4 in patients with tuberculosis was primarily observed in individuals with advanced involvement of lung parenchymal cells and high bacterial loads in the sputum.^[10]^ The IL-4 concentration may help healthcare providers diagnose and manage patients’ conditions. Over the past 20 years, several studies have reported a correlation between IL-4 levels and TB. Most researchers believe that increased serum IL-4 levels result from the immune response in patients with TB.^[11]^ However, Chukwuanukwu et al^[12]^ found that the level of IL-4 in individuals with TB was lower than that in healthy individuals. Chandrashekara et al,^[13]^ Bai et al,^[14]^ and others reported that the level of IL-4 was not significantly different between patients with TB and healthy individuals.

Thus far, the view that patients with TB have increased levels of IL-4 remains controversial. Saghazadeh and Rezaei^[15]^ conducted a systematic evaluation and meta-analysis of cytokine levels in the cerebrospinal fluid of patients with TB and controls. They found that the level of IL-4 in the cerebrospinal fluid of patients with tuberculous meningitis was high. However, that study mainly focused on tuberculous meningitis, and the incidence of pulmonary TB was higher than that of tuberculous meningitis. Therefore, the relationship between IL-4 levels and TB needs to be verified through observational studies. Zeng et al^[16]^ conducted a meta-analysis of Th1 and Th2 cytokine profiles to differentiate tuberculous from malignant pleural effusions. However, the main participants of this study were patients with tuberculous pleurisy and malignant pleural effusion. Recent studies have confirmed that high IL-4 levels are prevalent in patients with TB, particularly those with active pulmonary TB. Thus, this meta-analysis aimed to include the latest observational studies to determine whether patients with TB exhibit high IL-4 levels.

## 2. Materials and methods

### 2.1. Search strategy and literature selecting

This meta-analysis was registered in the International Prospective Register of Systematic Evaluations (https://www.crd.york.ac.uk/PROSPERO/, ID CRD42022365237). A researcher comprehensively searched the articles published in PubMed, EMBASE, VIP, Web of Science, China National Knowledge Infrastructure, and Wan Fang databases from January 1980 to October 2022 with the keywords (“tuberculosis” or “TB”) and (“Interleukin-4” or “IL-4”). The search formulas and information are listed in Table S1, Supplemental Digital Content, http://links.lww.com/MD/J144.

Criteria for inclusion: Case-control study, cross-sectional study, or cohort study; the content of literature research involved the level of IL-4 in blood samples or other body fluids of patients with TB; patients with TB included pulmonary TB, extrapulmonary TB, active TB, latent TB; the content of the literature was not limited by age, gender, nationality, or race; laboratory-based quantitative analysis was used to measure the level of IL-4 in patients with TB and non-TB controls; the parameters for the diagnosis of active pulmonary TB were the following^[17]^: symptoms that were typically associated with TB, computed tomography, an X-ray of the chest and positive sputum culture. Latent TB was diagnosed according to criteria previously established in the literature^[18]^; the literature data were complete, or the required data could be calculated according to known results; the IL-4 concentration was in the mean data format and standard deviation or formats that could be changed to this expression form; the search was limited to full-text articles; and for research repeatedly published, with a large sample size or more detailed information.

The exclusion criteria were as follows: included patients with TB with other complications, such as human immunodeficiency virus (HIV), hepatitis C virus, tumors, and autoimmune diseases; absence of detailed information in the original article or inability to contact the research author to provide more detailed data; case reports, conference papers, meta-analyses, review articles, and animal studies; and duplicate publications.

### 2.2. Document selection, data extraction, and quality evaluation

Based on the above retrieval method, 2 researchers preliminarily selected eligible articles based on their titles and abstracts. Subsequently, articles that met the inclusion criteria were reevaluated by 2 researchers through careful reading and a thorough review of the full text. If 2 researchers did not agree, a third researcher was consulted, and a final consensus decision was made.

### 2.3. Data extraction and management

The relevant data were obtained from eligible articles and are presented in tables that include the following information: basic information, such as first author and publication date; and baseline characteristics of the participants of the study, such as country, race, age, sample size, study design, measurement method, measurement sample type, location of TB infection, the status of TB infection, TB drug resistance, and IL-4 concentration.

### 2.4. Quality evaluation

The Newcastle-Ottawa Scale was used to evaluate literature quality.^[19]^ In the case of any differences in the quality evaluation process, negotiation by the 2 reviewers or arbitration by experts in the area was the preferred means of reaching a solution. The highest total score for each study was 9 points, including comparability (1 item, full score of 2 points), study population selection (4 items, full score of 4 points), and exposure or results (3 items, full score of 3 points). Scores of 0–3, 4–6, and 7–9 represent low, medium, and high quality, respectively.^[20]^

### 2.5. Ethical review

Ethical approval and patient consent were not required because the meta-analysis and bioinformatic analyses were based on published research and public databases.

### 2.6. Statistical treatment

Summarized data collected from the eligible studies were examined in this meta-analysis using STATA version 11.0 (Stata Corporation, College Station, TX). Continuous outcomes after harmonization were depicted as standard mean differences (SMDs) with 95% confidence intervals (95% CIs). Values of *P* > .1 and *I*^2^ < 50% depicted non-significant heterogeneity for which a fixed-effects model was selected. In comparison, a random effects model was used when *P* < .1 along *I*^2^ > 50% indicated heterogeneity among the data. The data heterogeneity was examined using subgroup analysis, descriptive analysis, or meta-regression. The ethnicity, study design, drug-resistant TB, infection site, infection status, and detection methods were examined using subgroup analyses. After eliminating 1 study at a time, a sensitivity test was conducted to examine the effect of each on the pooled SMD. Quantitative Begg’s and Egger’s tests were used to investigate publication bias.

## 3. Results

### 3.1. Literature retrieval and inclusion

**Figure 1.**
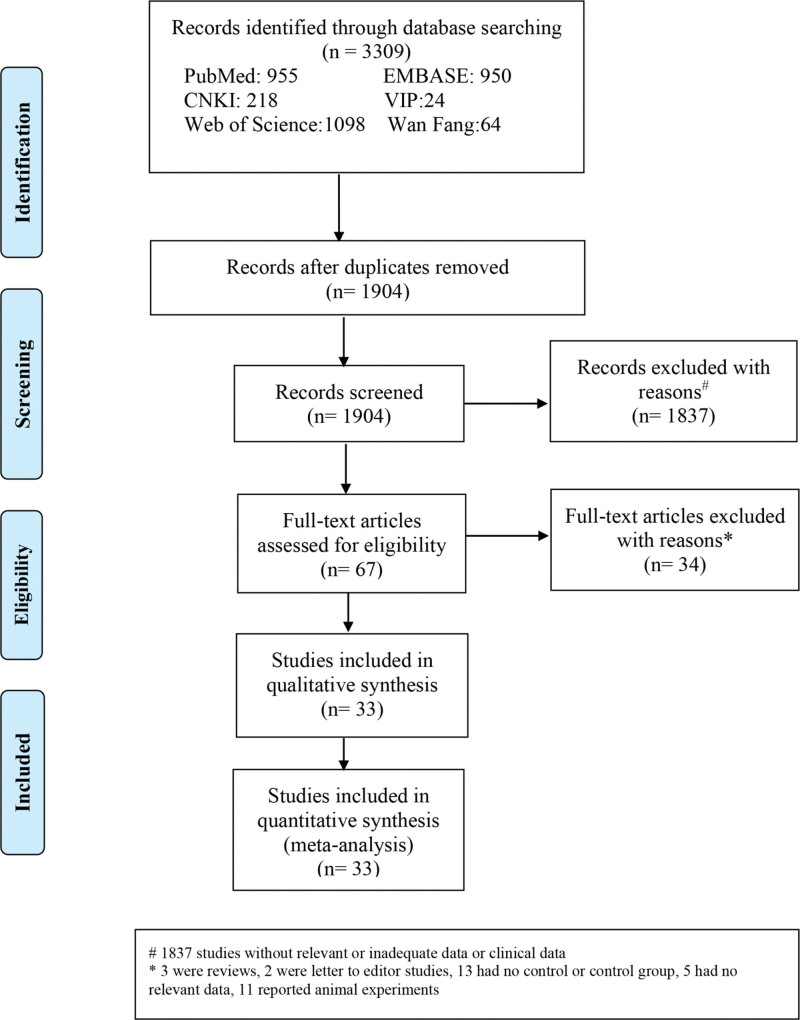
Selection process flowchart for search results and studies.

A total of 3309 Chinese and English articles were retrieved, of which 1904 were excluded after eliminating repeated studies through preliminary reading. Rereading and reviewing the titles and abstract excluded 1837 articles, resulting in 67 articles. The 67 articles were downloaded, and the text was thoroughly reviewed. According to the inclusion and exclusion criteria, 34 articles were excluded. In total, 33 articles were included, including three in Chinese^[21–23]^ and 30 in English.^[9,11,12,14,24–49]^ The literature retrieval process is illustrated in Figure 1.

### 3.2. Basic characteristics and quality of included documents

Among the 33 articles included,^[9,11,12,14,21–49]^ 51 studies were included, comprising 2379 patients with TB and 1391 healthy adults, including 2113 patients with pulmonary TB, 266 with extrapulmonary TB, 25 with multidrug-resistant TB, 2000 with active TB, and 379 with latent TB. Tables 1 and 2 provide basic information about the included studies.

**Table 1:** Characteristics of included studies.

Study	Year	Country	Ethnicity	NOS	Study design	Assay approach	Sample type	Infection site	Drug resistance	Infection status	Sample	
											TB	HC
Abedini A	2020	Iran	Caucasian	6	CCS	ELISA	Serum	PTB	Mixed	Active	24	30
Abhimanyu (EP-TB)	2016	India	African	7	CS	ELISA	Serum	LNTB	Mixed	Active	50	84
Abhimanyu (PTB)	2016	India	African	7	CS	ELISA	Serum	PTB	Mixed	Active	84	84
Ashenafi S (BAL)	2014	Ethiopia	African	7	CCS	ELISA	BAL	PTB	Mixed	Active	22	10
Ashenafi S (Plasma)	2014	Ethiopia	African	7	CCS	ELISA	Plasma	PTB	Mixed	Active	22	10
Bai R (Active PTB)	2019	China	Asian	7	CCS	ELISA	Serum	PTB	Mixed	Active	46	30
Bai R (LTB)	2019	China	Asian	7	CCS	ELISA	Serum	PTB	Mixed	Latent	38	30
Bai XJ	2018	China	Asian	7	CCS	ELISA	Serum	PTB	Mixed	Active	215	29
Baliko Z	1998	Hungary	Caucasian	6	CCS	ELISA	Serum	PTB	Mixed	Active	13	11
Benjamin R	2013	India	African	7	CCS	ELISA	Plasma	PTB	Mixed	Active	57	57
Chukwuanukwu RC (Active PTB)	2016	Britain	Caucasian	8	CCS	ELISA	Serum	PTB	Mixed	Active	41	35
Chukwuanukwu RC (LTB)	2016	Britain	Caucasian	8	CCS	ELISA	Serum	PTB	Mixed	Latent	30	35
Cui HY	2010	China	Asian	7	CCS	ELISA	Serum	Pleurisy TB	Without MDR TB	Active	36	30
Deveci F (Active PTB)	2005	Turkey	Caucasian	6	CCS	ELISA	Serum	PTB	Without MDR TB	Active	20	9
Deveci F (LTB)	2005	Turkey	Caucasian	6	CCS	ELISA	Serum	PTB	Without MDR TB	Latent	9	9
Dubaniewicz A	2007	Poland	Caucasian	8	CSS	CBA	Plasma	PTB	Mixed	Active	20	20
Huang SF (Active PTB)	2019	China	Asian	7	CS	ELISA	Serum	PTB	Mixed	Active	25	23
Huang SF (LTB)	2019	China	Asian	7	CS	ELISA	Serum	PTB	Mixed	Latent	32	23
Kart L	2003	Turkey	Caucasian	7	CSS	ELISA	Serum	PTB	Mixed	Active	43	19
kathamuthu GR (Active PTB)	2021	America	Caucasian	7	CS	ELISA	Plasma	PTB	Mixed	Active	44	44
kathamuthu GR (LTB)	2021	America	Caucasian	7	CS	ELISA	Plasma	PTB	Mixed	Latent	44	44
Korma W (Active EP-TB)	2020	Ethiopia	African	7	CCS	ELISA	Plasma	LNTB	Mixed	Active	23	51
Korma W (Active PTB)	2020	Ethiopia	African	7	CCS	ELISA	Plasma	PTB	Mixed	Active	35	51
Korma W (LTB)	2020	Ethiopia	African	7	CCS	ELISA	Plasma	PTB	Mixed	Latent	42	51
Kumar NP (EP-TB)	2013	India	African	7	CSS	ELISA	Plasma	EP-TB*	Mixed	Active	22	19
Kumar NP (PTB)	2013	India	African	7	CSS	ELISA	Plasma	PTB	Mixed	Active	14	19
Li BX (Active PTB)	2012	China	Asian	7	CCS	CBA	Serum	PTB	Mixed	Active	46	30
Li BX (LTB)	2012	China	Asian	7	CCS	CBA	Serum	PTB	Mixed	Latent	38	30
Liu Q (MTB)	2018	China	Asian	7	CCS	ELISA	CSF	MTB	Mixed	Active	43	11
Liu QY	2018	China	Asian	6	CCS	ELISA	Serum	PTB	Mixed	Active	124	52
Mendez A (MTB)	2010	Britain	Caucasian	8	CCS	ELISA	Serum	MTB	Mixed	Active	19	29
Mendez A (PTB)	2010	Britain	Caucasian	8	CCS	ELISA	Serum	PTB	Mixed	Active	34	29
Mendez A (RTB)	2010	Britain	Caucasian	8	CCS	ELISA	Serum	RTB	Mixed	Active	21	29
Moideen K (Active PTB)	2020	India	African	7	CCS	ELISA	Plasma	PTB	Mixed	Active	44	44
Moideen K (LTB)	2020	India	African	7	CCS	ELISA	Plasma	PTB	Mixed	Latent	44	44
Mustafa T	2015	Norwegian	Caucasian	8	CCS	ELISA	Serum	LNTB	Mixed	Active	61	23
Sharma RK	2016	India	African	7	CCS	ELISA	Vitreous fluid	Uveitis TB	Mixed	Active	17	18
Stalenhoef JE	2008	India	African	6	CSS	ELISA	Serum	PTB	Mixed	Active	34	36
Sun H	2022	China	Asian	6	CCS	ELISA	Serum	PTB	Mixed	Active	54	27
Sun XL (Active PTB)	2022	China	Asian	6	CS	ELISA	Serum	PTB	Mixed	Active	63	62
Sun XL (LTB)	2022	China	Asian	6	CS	ELISA	Serum	PTB	Mixed	Latent	63	62
Talat N	2011	Pakistan	Caucasian	7	CS	CBA	Plasma	PTB	Mixed	Active	19	37
Tan Q (MDR TB)	2012	China	Asian	6	CCS	CBA	Serum	PTB	MDR TB	Active	25	25
Tan Q (Without MDR TB)	2012	China	Asian	6	CCS	CBA	Serum	PTB	Without MDR TB	Active	23	25
Thillai M (BAL)	2012	Britain	Caucasian	7	CCS	ELISA	BAL	PTB	Mixed	Active	12	16
Thillai M (Serum)	2012	Britain	Caucasian	7	CCS	ELISA	Serum	PTB	Mixed	Active	15	40
Wang L	2002	China	Asian	6	CCS	ELISA	Serum	PTB	Mixed	Active	124	100
Yan LP (Active PTB)	2013	China	Asian	7	CSS	ELISA	Serum	PTB	Mixed	Active	43	40
Yan LP (LTB)	2013	China	Asian	7	CSS	ELISA	Serum	PTB	Mixed	Latent	39	40
Zhang J	2016	China	Asian	7	CCS	ELISA	Serum	PTB	Mixed	Active	48	20
Zhao Y	2021	China	Asian	7	CCS	ELISA	Serum	PTB	Mixed	Active	280	280

BAL = bronchoalveolar lavage, CBA = cytometric bead array, CCS = case–control study, CS = cohort study, CSS = cross sectional study, ELISA = enzyme linked-immuno-sorbent assay, EP-TB = extrapulmonary tuberculosis, HC = healthy controls, LNTB = tubercular lymphadenitis, LTB = latent tuberculosis infection, MDR TB = multidrug-resistant tuberculosis, MTB = meningeal tuberculosis, NA = not applicable, NOS = Newcastle-Ottawa Scale, PTB = pulmonary tuberculosis, RTB = renal tuberculosis, TB = tuberculosis.

* Including meningeal tuberculosis and spinal tuberculosis, abdominal tuberculosis (including peritonitis or tuberculoma) and tubercular lymphadenitis.

**Table 2:** Participants’ characteristics of included studies.

Study	Year	Sample(n)	Age		IL-4		
		TB/HC	TB	HC	TB	HC	Unit
Abedini A	2020	24/30	55.25 ± 21.58	52.2 ± 12.54	12.44 ± 2.06	2.48 ± 0.55	pg/mL
Abhimanyu (EP-TB)	2016	50/84	25 ± 1.7	27 ± 5	3.4 ± 2.1	4.5 ± 2.1	pg/mL
Abhimanyu (PTB)	2016	84/84	32 ± 2.6	27 ± 5	8.5 ± 12	4.5 ± 2.1	pg/mL
Ashenafi S (BAL)	2014	22/10	27.24 ± 8.11	28.49 ± 9.05	48.45 ± 62.37	15.2 ± 14.16	pg/mL
Ashenafi S (Plasma)	2014	22/10	27.24 ± 8.11	28.49 ± 9.05	50.68 ± 19.53	23.5 ± 17.02	pg/mL
Bai R (Active PTB)	2019	46/30	NA	NA	5.13 ± 1.32	4.87 ± 1.23	pg/mL
Bai R (LTB)	2019	38/30	NA	NA	5.02 ± 1.43	4.87 ± 1.23	pg/mL
Bai XJ	2018	215/29	34.1 ± 12.3	38.1 ± 7.3	0.94 ± 0.9	1.65 ± 0.81	pg/mL
Baliko Z	1998	13/11	47.1 ± 16	34.1 ± 9.4	5.76 ± 1.36	5.72 ± 1.48	pg/mL
Benjamin R	2013	57/57	NA	NA	20.26 ± 4.58	14.56 ± 2.66	pg/mL
Chukwuanukwu RC (Active PTB)	2016	41/35	36.5 ± 13.5	37.3 ± 11.6	28.77 ± 52.86	49.5 ± 43.3	pg/mL
Chukwuanukwu RC (LTB)	2016	30/35	36.3 ± 10.6	37.3 ± 11.6	5.5 ± 8.8	49.5 ± 43.3	pg/mL
Cui HY	2010	36/30	38 ± 17	45 ± 10	37.04 ± 13.34	31.66 ± 3.43	µg/mL
Deveci F (Active PTB)	2005	20/9	38.7 ± 18.51	33.7 ± 4.27	33.5 ± 2.6	33.9 ± 1.9	pg/mL
Deveci F (LTB)	2005	9/9	35.67 ± 18.02	33.7 ± 4.27	32.4 ± 2.2	33.9 ± 1.9	pg/mL
Dubaniewicz A	2007	20/20	45.9 ± 16.7	33.9 ± 11	6.5 ± 1.26	9 ± 1.7	pg/mL
Huang SF (Active PTB)	2019	25/23	56.3 ± 5.5	48.9 ± 4.3	0.27 ± 0.1	0.36 ± 0.1	µg/dL
Huang SF (LTB)	2019	32/23	45 ± 3.14	48.3 ± 4.3	0.1 ± 0.03	0.36 ± 0.1	µg/dL
Kart L	2003	43/19	36.4 ± 15.5	34.9 ± 12.7	0.5 ± 1	0.3 ± 0.3	pg/mL
kathamuthu GR (Active PTB)	2021	44/44	28.4 ± 7.5	36.7 ± 8.4	178 ± 25.46	64.6 ± 17.88	pg/mL
kathamuthu GR (LTB)	2021	44/44	37.4 ± 9.8	36.7 ± 8.4	36.57 ± 10.86	64.6 ± 17.88	pg/mL
Korma W (Active EP-TB)	2020	23/51	NA	NA	78.98 ± 59.44	75.35 ± 48.46	pg/mL
Korma W (Active PTB)	2020	35/51	NA	NA	82.33 ± 58.21	75.35 ± 48.46	pg/mL
Korma W (LTB)	2020	42/51	NA	NA	60.88 ± 50.86	75.35 ± 48.46	pg/mL
Kumar NP (EP-TB)	2013	22/19	7.4 ± 3.4	8.8 ± 3.5	7.02 ± 1.98	9.45 ± 2.36	pg/mL
Kumar NP (PTB)	2013	14/19	8.6 ± 4	8.8 ± 3.5	8.35 ± 2.12	9.45 ± 2.36	pg/mL
Li BX (Active PTB)	2012	46/30	34.8 ± 11	35.8 ± 9.5	5 ± 1.6	4.6 ± 0.5	pg/mL
Li BX (LTB)	2012	38/30	43.2 ± 14.2	35.8 ± 9.5	4.8 ± 1.6	4.6 ± 0.5	pg/mL
Liu Q (MTB)	2018	17/11	36.9 ± 11.7	50.19 ± 6.9	59.06 ± 39.82	5.07 ± 2.66	pg/mL
Liu QY	2018	124/52	NA	NA	318.26 ± 27.69	150.04 ± 20.88	ng/mL
Mendez A (MTB)	2010	19/29	NA	NA	54.23 ± 13.17	47.47 ± 17.78	pg/mL
Mendez A (PTB)	2010	34/29	NA	NA	48.13 ± 24.55	47.47 ± 17.78	pg/mL
Mendez A (RTB)	2010	21/29	NA	NA	60.07 ± 65.21	47.47 ± 17.78	pg/mL
Moideen K (Active PTB)	2020	44/44	39.3 ± 7.9	36.7 ± 8.4	5.02 ± 3.36	2.27 ± 2.89	pg/mL
Moideen K (LTB)	2020	44/44	37.4 ± 9.8	36.7 ± 8.4	3.1 ± 3.6	2.27 ± 2.89	pg/mL
Mustafa T	2015	61/23	30.7 ± 12.7	NA	1.43 ± 3.99	0.59 ± 1.11	pg/mL
Sharma RK	2016	17/18	NA	NA	8.41 ± 4.25	7.46 ± 5.41	pg/mL
Stalenhoef JE	2008	34/36	43 ± 7.1	45 ± 8.6	164.84 ± 38.69	136.67 ± 48.64	pg/mL
Sun H	2022	54/27	NA	NA	180 ± 18	121 ± 9	pg/mL
Sun XL (Active PTB)	2022	63/62	NA	NA	80.36 ± 25.64	51.06 ± 12.68	ng/L
Sun XL (LTB)	2022	63/62	NA	NA	56.33 ± 15.87	51.06 ± 12.68	ng/L
Talat N	2011	19/37	31.53 ± 13.3	24.81 ± 14.7	10.06 ± 5.01	4.6 ± 7.3	pg/mL
Tan Q (MDR TB)	2012	23/25	42.9 ± 11.7	41.1 ± 7.4	6.62 ± 4.64	3.72 ± 1.61	pg/mL
Tan Q (Without MDR TB)	2012	25/25	39.7 ± 11.4	41.1 ± 7.4	82.87 ± 32.69	3.72 ± 1.61	pg/mL
Thillai M (BAL)	2012	12/16	43.4 ± 16.2	28 ± 6.78	1.9 ± 0.71	0.33 ± 0.54	pg/mL
Thillai M (Serum)	2012	15/40	38.16 ± 13.22	32.8 ± 9.3	0.15 ± 0.23	2.97 ± 3.09	pg/mL
Wang L	2002	124/100	51 ± 4	49 ± 9	0.439 ± 0.16	0.421 ± 0.024	pg/mL
Yan LP (Active PTB)	2013	43/40	43 ± 15	40 ± 15	60.1 ± 39	50.4 ± 25	ng/L
Yan LP (LTB)	2013	39/40	39 ± 17	40 ± 15	91 ± 57.9	50.4 ± 25	ng/L
Zhang J	2016	48/20	51.54 ± 14.36	45.4 ± 16.6	2.08 ± 0.37	1.8 ± 0.15	pg/mL
Zhao Y	2021	280/280	55.6 ± 15.93	53.39 ± 14.77	2.68 ± 0.55	1.62 ± 0.17	pg/mL

BAL = bronchoalveolar lavage, EP-TB = extrapulmonary tuberculosis, HC = healthy controls, LTB = latent tuberculosis infection, MDR TB = multidrug-resistant tuberculosis, MTB = meningeal tuberculosis, NA = not applicable, PTB = pulmonary tuberculosis, RTB = renal tuberculosis, sIL-4 = interleukin-4, TB = tuberculosis.

### 3.3. Document quality evaluation

The quality of the assembled data was assessed using the Newcastle-Ottawa Scale.^[19]^ The results showed that each article in this study had a score of more than 6 points, including 5 articles with 8 points, 16 with 7 points, and 12 with 6 points, indicating the relatively high quality of the included literature (see Table 1).

### 3.4. Meta-analysis

#### 3.4.1. Overall analysis

**Figure 2.**
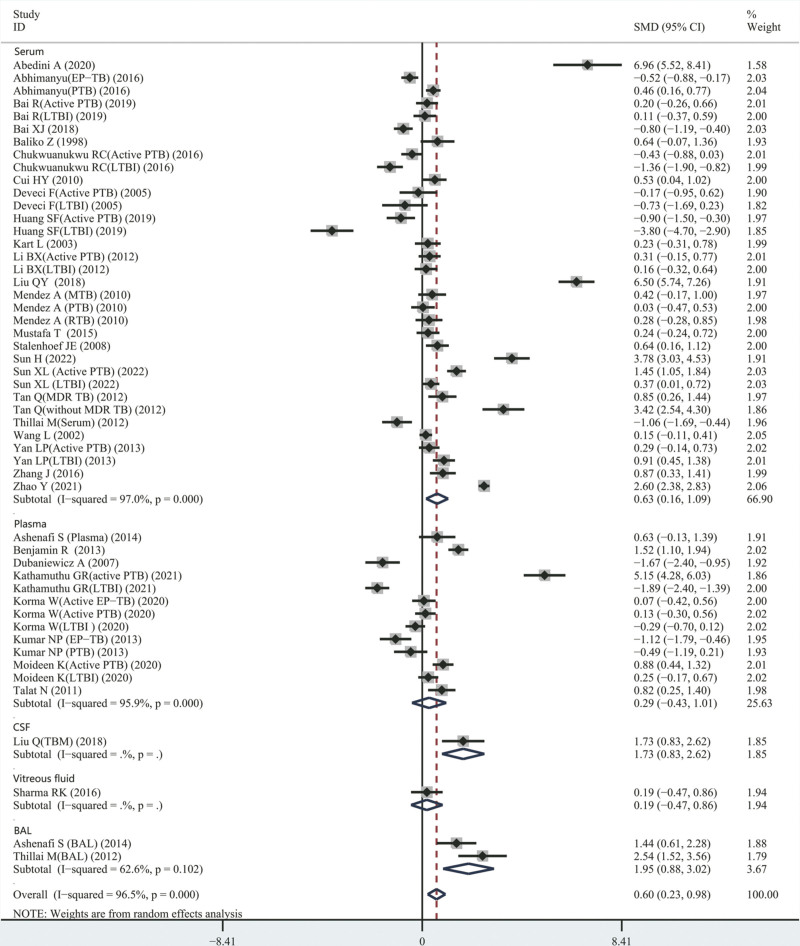
SMD forest plot and its 95% CI for IL-4 concentration level in the TB group and the healthy control group. CI = confidence interval, IL-4 = interleukin-4, SMD = standard mean differences, TB = tuberculosis.

The 33 articles included 51 studies.^[9,11,12,14,21–49]^ The 51 studies included 4317 subjects in total, and there was heterogeneity between the results of each study (χ^2^ = 1432.49, *I*^2^ = 96.5%, *P* < .0001); therefore, the random effects model was employed for the combined analysis. Meta-analysis results depicted that the concentration of IL-4 in patients with TB was significantly increased than in the control group (SMD = 0.60, [95% CI, 0.23–0.98], *P* = .002) as depicted in Figure 2. As variations in the sample types could be linked to an associated increase in heterogeneity, the relationship between serum IL-4 and plasma IL-4 levels and TB was analyzed, as shown in Table 3. The number of articles linked to the level of IL-4 in cerebrospinal fluid, lavage fluid, or vitreous fluid was small; thus, we conducted descriptive analyses of these studies.

**Table 3:** Subgroup analyses of IL-4 levels in TB and controls.

Subgroup analysis of plasma levels (n)	SMD [95% CI], *P* value, *I*^2^ (%), *P*_h_	Subgroup analysis of serum levels (n)	SMD [95% CI], *P* value, *I*^2^ (%), *P*_h_
Overall (13)	0.290 [−0.430 to 1.010], .429, 95.9%, <.0001	Overall (34)	0.630 [0.162, 1.092], .010, 97.0%, <.0001
**Ethnicity**		**Ethnicity**	
Caucasian (4)	0.589 [−2.14, 3.318], .672, 98.6%, <.0001	Caucasian (12)	0.224 [−0.396, 0.844], .479, 91.8%, <.0001
African (9)	0.194 [−0.303, 0.691], .444, 88.9%, <.0001	Asian (19)	0.887 [0.202, 1.573], .011, 97.8%, <.0001
**Study design**		African (3)	0.185 [−0.528, 0.898], .610, 90.8%, <.0001
CCS (7)	0.453 [−0.036, 0.941], .069, 87.2%, <.0001	**Study design**	
CSS (3)	−1.089 [−1.744, −0.433], .001, 62.5%, .070	CCS (24)	0.906 [0.272, 1.540], .005, 97.5%, <0.0001
CS (3)	1.345 [−2.223, 4.913], .460, 99.0%, <.0001	CSS (4)	0.527 [0.215, 0.839], .001, 41.4%, .163
**Assay approach**		CS (6)	−0.421 [−1.344, 0.503], .372, 96.6%, <.0001
ELISA (11)	0.416 [−0.374, 1.206], .302, 96.2%, <.0001	**Assay approach**	
CBA (2)	−0.413 [−2.858, 2.032], .470, 96.4%, <.0001	ELISA (30)	0.541 [0.032, 1.050], .037, 97.2%, <.0001
**Infection site**		CBA (4)	1.127 [0.028, 2.225], .044, 93.3%, <.0001
PTB (11)	0.440 [−0.385, 1.265], .296, 96.4%, <.0001	**Infection site**	
EP-TB (2)	−0.505 [−1.673, 0.663], .397, 87.5%, .005	PTB (29)	0.689 [0.152, 1.226], .012, 97.3%, <.0001
**Infection status**		EP-TB (5)	0.166 [−0.275, 0.606], .461, 75.7%, .002
Active (10)	0.579 [−0.274, 1.431], .183, 95.6%, <.0001	**Drug resistance**	
Latent (3)	−0.636 [−1.818, 0.546], .291, 95.3%, <.0001	MDR TB (1)	NA
		**Without MDR TB (4**)	0.762 [−0.778, 2.302], .332, 94.0%, <.0001
		Mixed (29)	0.584 [0.072, 1.095], .025, 97.3%, <.0001
		**Infection status**	
		Active (27)	0.920 [0.387, 1.452], .001, 97.2%, <.0001
		Latent (7)	−0.565 [−1.436, 0.305], .203, 94.7%, <.0001

BAL = bronchoalveolar lavage, CBA = cytometric bead array, CCS = case–control study, CI = confidence interval, CS = cohort study, CSS = cross sectional study, ELISA = enzyme linked-immuno-sorbent assay, EP-TB = extrapulmonary tuberculosis, MDR TB = multidrug-resistant tuberculosis, NA = not applicable, PTB = pulmonary tuberculosis.

#### 3.4.2. Comparison of serum IL-4 levels between patients with TB and healthy controls

In this meta-analysis, 34 studies^[9,11,12,14,21–39]^ investigated the variance in the concentrations of serum IL-4 in individuals with TB and healthy subjects. The results indicated an increased amount of IL-4 in the serum of the patients with TB than the other group (SMD = 0.630, [95% CI, 0.162–1.092], *I*^2^ = 97.0%, *P* = .01; see Fig. 2 and Table 3).

#### 3.4.3. Comparison of plasma IL-4 levels between patients with TB and healthy controls

In this review, 13 observational studies^[42–48]^ investigated the difference between the levels of IL-4 in the plasma of individuals with TB and healthy subjects. The analysis did not indicate any major statistical variation between the plasma IL-4 levels of the 2 groups (SMD = 0.290, [95% CI, −0.430 to 1.010], *I*^2^ = 95.9%, *P* = .429; see Fig. 2 and Table 3).

#### 3.4.4. Comparison of IL-4 concentration in the cerebrospinal fluid between patients with tuberculous meningitis and the control group

Liu et al^[49]^ found that the concentration of IL-4 in cerebrospinal fluid of tuberculous meningitis patients was higher than that of non-tuberculous meningitis patients (59.06 ± 39.82 pg/mL vs 5.07 ± 2.66 pg/mL, *P* < .05) by comparing 17 patients with tuberculous meningitis to patients with non-tuberculous meningitis.

#### 3.4.5. Comparison of IL-4 concentration in the vitreous fluid between patients with TB and the control group

Sharma et al^[41]^ quantified the concentration of IL-4 in the patient’s vitreous fluid by Enzymes Linked-Immuno-sorbent Assay method and found that the concentration of IL-4 in the patient’s vitreous fluid with tuberculous uveitis was higher than that in the patient with non-tuberculous uveitis (8.41 ± 4.25 pg/mL vs 7.46 ± 5.41 pg/mL, *P* < .05).

#### 3.4.6. Comparison of IL-4 concentration in alveolar lavage fluid between patients with TB and healthy controls

In the included literature, 2 studies^[38,40]^ compared the concentration of IL-4 in alveolar lavage fluid between patients with TB and healthy subjects. The results showed that the IL-4 concentration in the alveolar lavage fluid of individuals with patients with TB was higher than that in controls (SMD = 1.952, [95% CI, 0.883–3.022], *I*^2^ = 62.6%, *P* < .0001; see Fig. 2).

### 3.5. Comparison of IL-4 levels between active patients with TB and latent patients with TB

**Figure 3.**
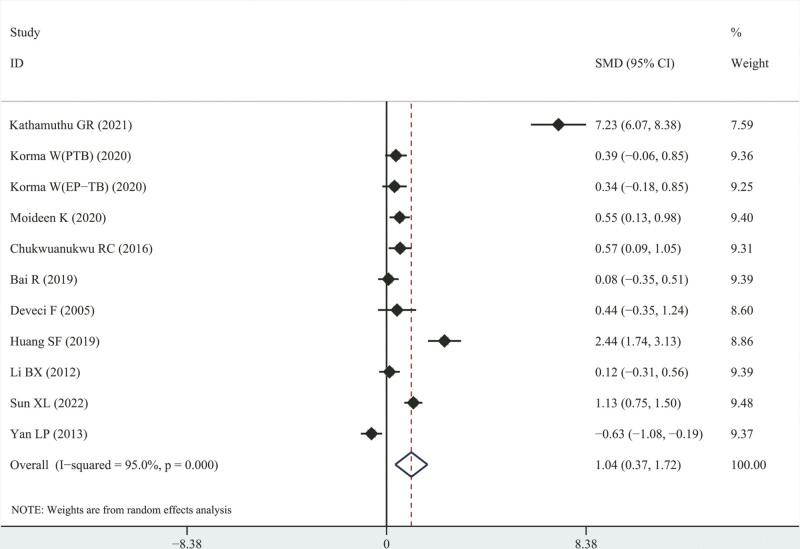
SMD forest plot and its 95% CI for IL-4 concentration level in the active tuberculosis group and active tuberculosis group. CI = confidence interval, IL-4 = interleukin-4, SMD = standard mean differences.

Eleven studies^[12,14,23,29,30,36,39,44–46]^ compared the variation in the degree of IL-4 concentration in individuals with latent and active TB. The results showed that IL-4 levels in individuals with active TB were higher than those in individuals with latent TB (SMD = 1.042, [95% CI, 0.366–1.719], *I*^2^ = 95.0%, *P* = .003; see Fig. 3).

### 3.6. Subgroup analysis

The heterogeneity of the meta-analysis was high (*I*^2^ = 96.5, *P* < .0001), and different races, research types, detection methods, infection sites, infection status, and other parameters may have affected the heterogeneity of the study. Consequently, subgroup analysis of serum and plasma IL-4 levels was conducted according to the above factors.

#### 3.6.1. Subgroup analysis of serum IL-4 level in patients with TB

##### 3.6.1.1. Infection status

The experimental groups were further divided into active tuberculosis (ATB) and latent tuberculosis (LTB) groups for subgroup analyses. Twenty-seven studies^[9,11,12,14,21–28,30–39]^ involved ATB. The analysis revealed an increased level of serum IL-4 in ATB subjects compared to that in controls (SMD = 0.920, [95% CI, 0.387–1.452], *I*^2^ = 97.2%, *P* = .001). Seven studies involved LTB.^[12,14,23,29,30,36,39]^ The results showed no significant statistical difference in the serum IL-4 levels between LTB subjects and the control group (SMD = −0.565, [95% CI, −1.436 to 0.305], *I*^2^ = 94.7%, *P* = .203; see Table 3).

##### 3.6.1.2. Location of TB focus

TB bacilli can infect various tissues. In addition to pulmonary TB, intestinal, pleural, spinal, renal, and extrapulmonary TB can occur. Some studies have reported a relationship between the serum IL-4 levels of pulmonary TB and extrapulmonary TB and healthy controls. Therefore, subgroup analysis was conducted according to different TB infection sites. The results showed that 5 studies^[25,28,32,33]^ provided data on IL-4 levels in individuals with extrapulmonary TB and in healthy subjects. The results showed that there was no statistical difference between the serum IL-4 levels of individuals with extrapulmonary TB and healthy people (SMD = 0.166, [95% CI, −0.275 to 0.606], *I*^2^ = 75.7%, *P* = .461); 29 studies^[9,11,12,14,21–27,29–32,34–39]^ report on the difference in serum IL-4 between pulmonary TB and healthy people. The analysis indicated an increased serum IL-4 level in pulmonary TB individuals compared to controls (SMD = 0.689, [95% CI, 0.152–1.226], *I*^2^ = 97.3%, *P* = .01; see Table 3).

##### 3.6.1.3. Resistance

One study^[37]^ reported serum IL-4 levels in individuals with multiple drug resistance-TB (without multidrug-resistant TB). The literature indicated an increased amount of IL-4 in individuals with multiple drug resistance TB than the control (82.87 ± 32.69 pg/mL vs 3.72 ± 1.61 pg/mL, 0.05). Four studies reported that the included population was drug-susceptible.^[28,29,37]^ The results showed that there was no significant difference between the levels of IL-4 in patients with drug-susceptible TB and the healthy control group (SMD = 0.762, [95% CI, −0.778 to 2.302], *I*^2^ = 94%, *P* = .332). In addition, 29 studies^[9,11,12,14,21–27,30–36,38,39]^ did not strictly distinguish whether the enrolled population was drug-resistant. These were classified as mixed-group studies. The results showed higher serum levels of IL-4 in individuals with TB in the mixed group than in the healthy control group (SMD = 0.584, [95% CI, 0.072–1.095], *I*^2^ = 97.3%, *P* = .025; see Table 3).

##### 3.6.1.4. Ethnicity

According to ethnic differences, the subjects were categorized into Caucasian, Asian, and African populations, and serum IL-4 levels in healthy controls and patients with TB were compared. The results showed that in the Asian population, the serum IL-4 level of patients with TB was higher than that of the control group (SMD = 0.887, [95% CI, 0.202–1.573], *I*^2^ = 97.8%, *P* = .011). There was no major variation in serum IL-4 levels between TB subjects and the control group in the Caucasian (SMD = 0.224, [95% CI, −0.396 to 0.844], *I*^2^ = 91.8%, *P* = .479) and African populations (SMD = 0.185, [95% CI, −0.528 to 0.898], *I*^2^ = 90.8%, *P* = .61; see Table 3).

##### 3.6.1.5. Research design type

Twenty-four studies were case-control studies.^[9,11,12,14,21–24,26–29,32,33,35,37,38]^ The results disclosed that the serum IL-4 level of individuals with TB was greater than that of the healthy control group (SMD = 0.906, [95% CI, 0.272–1.540], *I*^2^ = 97.5%, *P* = .005); The 4 studies were cross-sectional studies.^[31,34,39]^ The results were similar to the aforementioned findings, with increased serum IL-4 levels in diseased individuals (SMD = 0.527, [95% CI, 0.215–0.839], *I*^2^ = 41.4%, *P* = .001). Another 6 studies were cohort studies.^[25,30,36]^ The results indicated no significant variation between the serum IL-4 levels of patients with TB and those of the control group (SMD = −0.421, [95% CI, −1.344 to 0.503], *I*^2^ = 96.6%, *P* = .372; see Table 3).

##### 3.6.1.6. Test method

In 30 studies, the serum IL-4 concentration was detected using ELISA.^[9,11,12,14,21,22,24–35,38,39]^ A cytometric bead array (CBA) measurement method was used in 4 studies.^[23,37]^ Using ELISA or CBA to detect IL-4, the serum IL-4 concentration in TB subjects was found to be greater than that in healthy controls (SMD = 0.541, [95% CI, 0.032–1.050], *I*^2^ = 97.2%, *P* = .037; SMD = 1.127, [95% CI, 0.028–2.225], *I*^2^ = 93.3%, *P* = .044; see Table 3).

#### 3.6.2. Subgroup analysis of plasma IL-4 level in patients with TB

##### 3.6.2.1. Infection status

The experimental group in 10 studies^[40,42–48]^ included patients with active TB, while the control group comprised healthy subjects. The results showed no statistically significant difference between the plasma IL-4 levels of patients with active TB and those of the healthy group (SMD = 0.579, [95% CI, −0.274 to 1.431], *I*^2^ = 95.6%, *P* = .183). The experimental group in the 3 studies included patients with latent TB, whereas the control group consisted of healthy subjects.^[44–46]^ The results showed no significant difference between the plasma IL-4 levels of the patients with LTB and those of the control group (SMD = −0.636, [95% CI, −1.818 to 0.546], *I*^2^ = 95.3%, *P* = .291; see Table 3).

##### 3.6.2.2. Location of TB focus

Subgroup analysis was performed according to the different TB infection sites. Two studies^[45,47]^ provided data on plasma IL-4 levels in patients with extrapulmonary TB and in healthy subjects. The results showed that there was no statistical difference between the plasma IL-4 levels of patients with extrapulmonary TB and those of the healthy group (SMD = −0.505, [95% CI, −1.673 to 0.663], *I*^2^ = 87.5%, *P* = .397); 11 studies^[40,42–48]^ provided data on plasma IL-4 levels in patients with pulmonary TB and healthy subjects. The results showed no statistically significant difference between the plasma IL-4 levels in patients with pulmonary TB and those in the control group (SMD = 0.440, [95% CI, −0.385 to 1.265], *I*^2^ = 96.4%, *P* = .296; see Table 3).

##### 3.6.2.3. Ethnicity

Subgroup analyses were conducted based on ethnicity. The results showed no significant difference in IL-4 levels between Caucasian patients and the healthy control group (SMD = 0.589, [95% CI, −2.14 to 3.318], *I*^2^ = 98.6%, *P* = .672), and the same results were observed in African races (SMD = 0.194, [95% CI, −0.303 to 0.691], *I*^2^ = 88.9%, *P* = .444; see Table 3).

##### 3.6.2.4. Research design

Seven case-control studies were included.^[40,42,45,46]^ The analysis indicated that the level of IL-4 in the plasma of patients with TB had no significant statistical significance compared with the healthy control group (SMD = 0.453, [95% CI, −0.036 to 0.941], *I*^2^ = 87.2%, *P* = .069); the 3 studies were cross-sectional studies.^[44,48]^ The results showed that the level of IL-4 in the plasma of patients with TB was higher than that of healthy controls (SMD = −1.089, [95% CI, −1.744 to 0.433], *I*^2^ = 62.5%, *P* = .001). Three other studies^[43,47]^ were cohort studies, and the results showed that there was no statistically significant difference between the plasma IL-4 levels of patients with TB and the healthy control group (SMD = 1.345, [95% CI, −2.223 to 4.913], *I*^2^ = 99%, *P* = .46; see Table 3).

##### 3.6.2.5. Detection method

Eleven articles^[40,42,44–47]^ used the measurement method of ELISA to detect the concentration of plasma IL-4, and 2 articles used the measurement method of CBA.^[43,48]^ Subgroup analyses were performed using various detection methods. In the subgroup analysis of ELISA, there was no significant difference between the plasma IL-4 levels of patients with TB and healthy subjects (SMD = 0.416, [95% CI, −0.374 to 1.206], *I*^2^ = 96.2%, *P* = .302). In the CBA subgroup analysis, there was no significant difference between the plasma IL-4 levels of patients with TB and the control group of healthy people (SMD = −0.413, [95% CI, −2.858 to 2.032], *I*^2^ = 96.4%, *P* = .47; see Table 3).

### 3.7. Meta-regression

The *I*^2^ value of the meta-analysis of the general population was 96.5%, indicating a high degree of heterogeneity. Therefore, meta-regression was used to determine the potential sources of heterogeneity. The meta-regression data showed that race, research design method, measurement method, TB infection location, TB infection status, and drug resistance to TB were not sources of heterogeneity (see Table 4). Other unknown confounding factors may have affected the heterogeneity in this study.

**Table 4:** Meta-regression analysis of variables predicting serum and plasma levels of IL-4.

Factors	Sample	Coefficient	Standard error	95% CI	Adjusted *R*^2^	*P*
Ethnicity	Serum	0.186	0.566	−0.966 to 1.339	−0.029	.774
	Plasma	−0.195	0.548	−1.400 to 1.011	−0.086	.729
Study design	Serum	−0.662	0.428	−1.533 to 0.209	0.041	.123
	Plasma	0.217	0.615	−1.135 to 1.570	−0.087	.73
Assay approach	Serum	0.609	1.067	−1.564 to 2.783	−0.023	.572
	Plasma	−0.84	1.386	−3.890 to 2.211	−0.061	.557
Infection site	Serum	−0.515	0.968	−2.486 to 1.456	−0.025	.598
	Plasma	−0.969	1.374	−3.993 to 2.055	−0.048	.495
Drug resistance	Serum	−0.141	0.766	−1.702 to 1.420	−0.033	.855
	Plasma	NA	NA	NA	NA	NA
Infection status	Serum	−1.54	0.809	−3.187 to 0.107	0.078	.066
	Plasma	−1.225	1.139	−3.731 to 1.281	0.015	.305

CI = confidence interval, IL-4 = interleukin-4, NA = not applicable.

### 3.8. Sensitivity analysis

**Figure 4.**
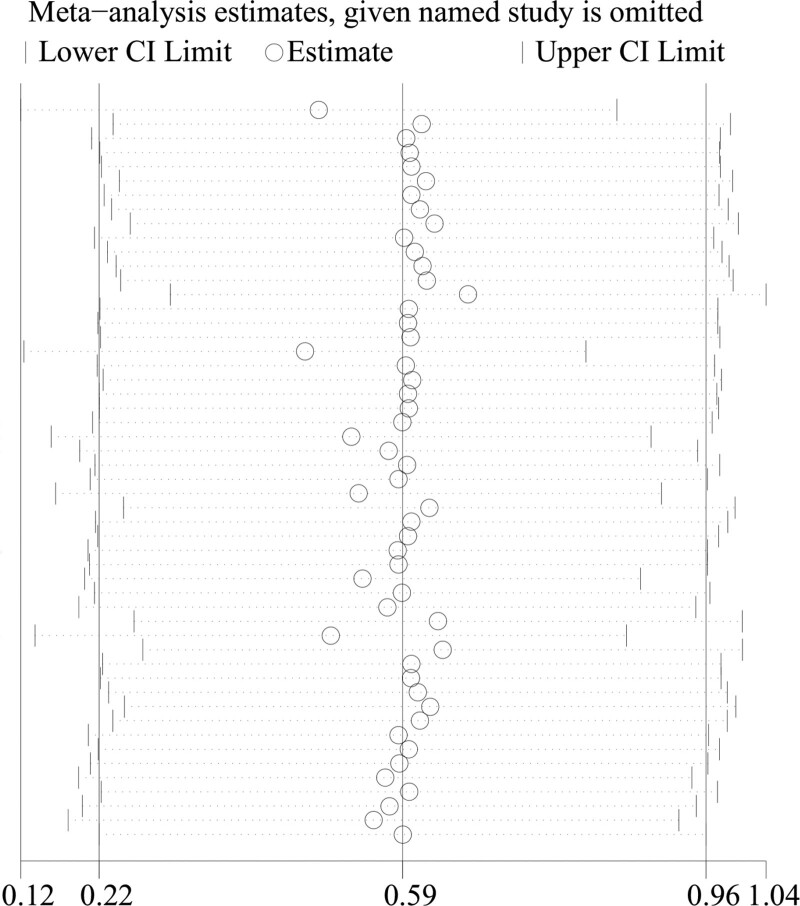
Sensitivity analysis of the overall pooled study.

A sensitivity analysis was performed to evaluate the stability of the meta-analysis. Statistically similar data were obtained after the sequential exclusion of each study, suggesting that our results were statistically reliable (see Fig. 4).

### 3.9. Publication of bias evaluation

**Figure 5.**
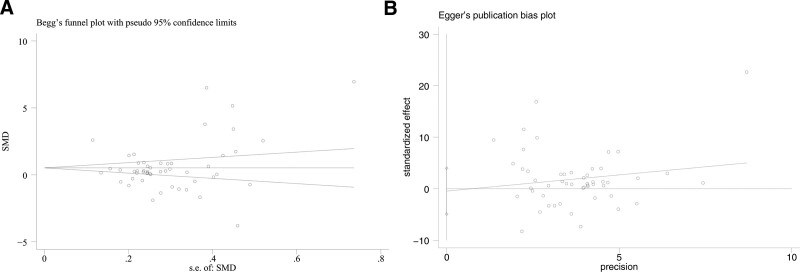
Begg’s test and Egger’s test were to evaluate the publication bias among those included literatures about the association between IL-4 concentration level and tuberculosis. (A) Begg’s test and (B) Egger’s test. IL-4 = interleukin-4.

The meta-analysis of total patients with TB used Stata 11.0 software for Begg’s and Egger’s tests, which depicted the absence of publication bias in this study (Begg’s test: *P* > .05; Egger’s test *t* = −0.21, *P* = .836; see Fig. 5A and B).

## 4. Discussion

**Figure 6.**
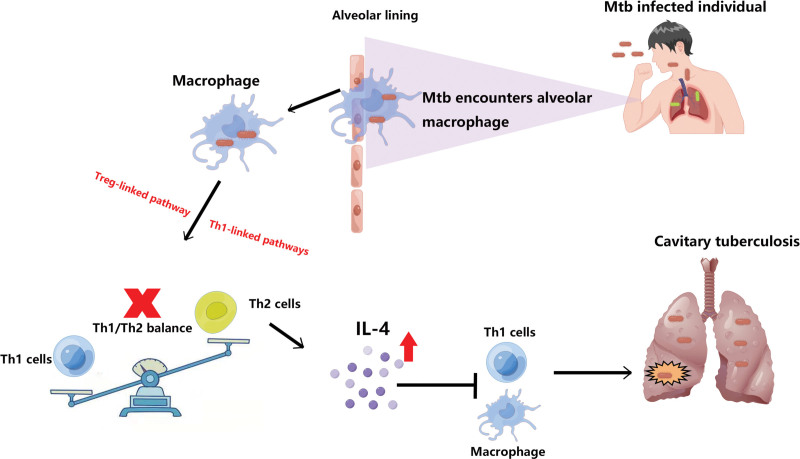
The possible potential mechanism of IL-4 aggravating the progression of tuberculosis. IL-4 = interleukin-4.

The number of Th1 and Th2 cells and the balance of Th1/Th2 play major roles in maintaining the body’s normal immune function and have an important impact on the onset and prognosis of TB.^[50]^ Once the Th1/Th2 balance is disrupted, it is likely to break the dynamic balance of the human cytokine network, causing disease.^[51]^ Studies have reported that after the body is infected with Mycobacterium TB, Th1 type immune response is suppressed, Th2 reactivity is increased, and Th1/Th2 imbalance ultimately leads to the progression of TB.^[52]^ IL-4 is mainly secreted by Th2 cells, and its main role is to stimulate the proliferation and differentiation of Th2 cells and promote the activation of B cells. In vitro research has shown that mycobacterial containment in human macrophages can be undermined by IL-4, likely through disrupting the pathways associated with Treg and Th1.^[53]^ Therefore, we proposed the following mechanistic hypothesis (Fig. 6).

This study is the first of its kind as it discusses the connection between the level of IL-4 and TB from the perspective of meta-analysis of IL-4. The level of IL-4 in individuals with TB was examined in healthy participants. A meta-analysis, subgroup analysis, and meta-regression analysis were performed, and various samples were utilized for quantifying IL-4, as well as other parameters that may influence the distribution of IL-4, such as race, research design type, and focus location. The main research results were as follows: in the absence of other respiratory diseases (including latent TB infection and non-TB infection), the serum/alveolar lavage fluid of individuals with TB contained considerably increased IL-4 concentrations, especially in the pulmonary tuberculosis group and active TB groups, while IL-4 levels in the plasma of individuals with pulmonary TB did not vary significantly from that of the healthy control group; IL-4 levels in serum may differ in Asian populations; and most of the studies on serum IL-4 levels of patients with TB were cross-sectional studies. In cross-sectional studies, the serum IL-4 level of patients with TB was greater than that of healthy controls; the IL-4 level in individuals with active TB was higher than that in those with latent TB; and the heterogeneity in the meta-analysis was not significantly affected by the location of TB infection, detection method, race, type of study design, or TB infection status.

Although the main finding of elevated blood levels of IL-4 in individuals with active pulmonary TB compared to healthy controls is reliable, a major portion of this heterogeneity remains to be elucidated. This meta-analysis was based on 51 observational studies, of which 28 reported higher IL-4 levels in patients with TB,^[9,11,21,22,24,28,32–37,39–42,44,46,48,49]^ 15 studies reported low levels of IL-4 in patients with TB,^[12,25,26,30,38,43,45,47]^ and 8 studies did not report any variation in the control and patient groups.^[14,23,27,29,31]^ First, an attempt was made to use a mixed healthy control group to separate latently infected and uninfected TB cases. But since most research did not take into account the diagnosis of latent TB infection in healthy control groups, this was difficult, and only a small number of studies solved this problem and provided relevant IL-4 data. Second, a subgroup analysis was conducted according to race, research design, detection method, TB infection site, drug resistance, and presence of active TB. Serum IL-4 levels were significantly different in specific subgroups of studies (Asian, pulmonary TB, cross-sectional studies, and active TB). It is worth noting that in Asians, the level of IL-4 in patients with TB is higher than that in the control group; however, in Caucasian and African populations, the level of IL-4 in patients with TB does not vary considerably from that in healthy controls. This indicates that race and genetic factors may also affect serum IL-4 levels in patients with TB. In a study by Kulprane et al,^[54]^ the allele distribution of IL-4 -590 C, -33 C, and VNTR R3 was greater among patients with pulmonary tuberculosis (PTB) (25.58%, 25.58%, and 25.58%, respectively) than among control subjects (20%, 20.48%, and 19.44%, respectively). This could indicate that IL-4-590C/T, -33C/T, and VNTR intron 3 may play a role in the susceptibility to PTB. However, Mansouri et al^[55]^ did not consider the difference in the allelic and genotypic frequencies of IL-4 as significant (-590C/T) between patients and controls. In addition, there may be a difference in the virulence of MTB strains prevalent in Asia, which could be the underlying reason. The meta-regression analysis showed that race, study design, infection site, detection method, and active TB were not sources of heterogeneity. However, these elements can potentially enhance heterogeneity and reduce the reliability of a meta-analysis. The “leave-one-out” sensitivity testing also showed that no specific research was responsible for the substantial heterogeneity. We hypothesized that additional factors might contribute to the heterogeneity, such as variations in the methods used for blood sampling and storage, techniques used for quantification, experimental conditions, and differences in nutrition, smoking, drinking, and other underlying confounding factors.

The potential role of IL-4 (a typical helper T cell type 2 (Th2) cytokine) in the pathophysiology of TB has been defined using various resources. The host response to M. host toward Mycobacterium TB infection is influenced by macrophages and their behavior.^[56]^ In animal models, mice with altered antibody profiles, inflammatory cytokines, and lung levels of tumor necrosis factor and stat1 are adversely affected by IL-4-responsive B cells during the chronic phase of tuberculosis.^[57]^ A recent increase in the conversion of cortisone into cortisol in tuberculous lesions may be responsible for elevated IL-4 levels, which correspond to disease severity, serum levels of IgE, and soluble CD30.^[58]^ Nolan et al^[59]^ reported that A potential biomarker for acid-fast bacilli in the sputum of patients might be the increased release of IL-4 by bronchoalveolar lavage in radiographically advanced TB. These most likely represent the ineffective immunological regulation of TB control mechanisms, resulting in increasingly infected patients. This evidence led us to conclude that the IL-4 levels in patients with TB are subject to changes. The level of IL-4 in the serum and lavage fluid of patients with TB was higher than that in the healthy control group, although there was no significant difference between the level of IL-4 in the plasma of patients with TB and that of the healthy control group, which may be due to the lack of research involving plasma samples. In addition, serum and plasma are the 2 major blood components, and coagulation factors from plasma sources may affect IL-4 expression.^[60]^ The results showed variations in the serum and plasma concerning protein concentration, which could be attributed to eliminating some cytokine sources in the plasma, as depicted by the diagnostic differences for IL-4 in the serum but not in the plasma.^[61]^ In addition, Liu et al^[49]^ found that the concentration of IL-4 in the cerebrospinal fluid of patients with tuberculous meningitis was higher than that in healthy patients, by comparing the data of 17 patients with 11 controls. Similarly, Sharma et al compared the levels of IL-4 in the vitreous fluid of 17 patients with tuberculous uveitis and 18 patients without, and found that the level of IL-4 in the vitreous fluid of patients with TB was higher than that in those without. Therefore, IL-4 plays a major role in human immunological reactions to Mycobacterium TB.

Clinical studies have suggested that IL-4 can be used as a biological marker to evaluate disease severity and activity.^[58]^ Compared to inactive, obsolete, or cured control groups, individuals with active pulmonary TB have increased levels of IL-4.^[62]^ Additionally, the concentration of IL-4 in positive sputum smears and culture results was higher than that in negative sputum smears and culture results.^[12]^ These meta-analyses also suggested that the level of IL-4 in patients with active TB was higher than that in patients with latent TB. Interferon and protective Th1 responses may be downregulated by the production of IL-4.^[63]^ Additionally, IL-4 activates macrophages, Toll-like receptor 2, and nitric oxide synthase in patients with PTB. These outcomes may affect the course of MTB infection and are crucial for preserving a stable environment for anti-MTB activity.^[14]^

In patients with pulmonary TB, the increase in circulating IL-4 is more significant in those with cavity-type and bilateral involvement.^[64]^ Most studies included in this review investigated the link between pulmonary TB and serum IL-4 levels. Therefore, the combined analysis results also showed that the serum IL-4 level of patients with pulmonary TB was greater than that of healthy controls; for extrapulmonary TB, due to the small sample size, the results of the combined study suggest that variation in serum IL-4 levels between the extrapulmonary TB and healthy control groups does not exist. However, some studies have shown that among patients with tuberculous pleurisy, those exhibiting pleural effusion also show higher levels of IL-4 than those with no pleural effusion, while higher levels of IL-4 may result in residual pleural thickening in patients.^[16]^ The study showed that the expression of interferon-γ in experimental mice was increased during early infection with Mycobacterium TB, while the formation of IL-4 was increased in the course of the chronic infection stage signified by progressive fibrosis and necrotic cavities.^[64]^ Prior research has documented no differences in serum IL-4 levels between individuals with active pulmonary TB and controls.^[65]^ Later studies showed that tissue necrosis was significantly influenced by an increase in IL-4 and CD8^+^ T cells among CD4^+^ T cells in patients with TB, particularly in those with cavitary TB.^[66]^ People in developing countries are at risk of exposure to high doses of Mycobacterium TB, which may cause sufficient IL-4 to damage Th1 cell effectors, leading to the spread of TB. In addition, studies have shown that the circulating IL-4 level in patients with drug-resistant TB is also higher than the baseline level, which may make it easier to prolong the negative conversion of sputum culture.^[37]^

The development of IL-4-mediated immune-related drugs may help balance the immune function of patients with TB and increase the efficacy of standard ATT. Roy et al^[67]^ revealed that a considerable reduction in bacterial load might be achieved by administering an anti-IL-4 antibody as a pulse during the early or late phases of mouse infection. Dheda et al^[68]^ found that the stability of mRNA encoding IL-4 was increased in pulmonary tuberculosis, whereas the stability of mRNA encoding the antagonistic splice variant IL-4delta2 was not. Dupilumab is a humanized monoclonal IgG4 antibody that suppresses IL-4 and IL-13 signaling by binding to the alpha subunit of the IL-4 receptor (IL-4R). It has also been used to treat atopic dermatitis and asthma. This function was further elaborated by Chen S, wherein the combined utilization of dupilumab in the form of an add-on treatment maintained disease control in individuals with refractory pemphigus vulgaris and pulmonary TB.^[69]^ It was previously reported that there was a considerable variation in individuals with TB and healthy subjects concerning IL-4 production, as the diseased individuals depicted a Th2 immune response mode, while TB-positive healthy people showed a Th1 mode in vitro.^[70]^ Surcel et al^[71]^ observed a similar phenomenon. They concluded that the production of IL-4 could be linked to the loss of the host protective response and, therefore, to the pathogenesis of TB. If this is true, it is easy to assume, as was the case for an experimental mouse model,^[67]^ that anti-IL-4 therapy may be used to treat patients with advanced diseases, especially those infected with multidrug-resistant bacteria. It is possible that an antagonist of disease-promoting cytokines might be useful in enhancing host resistance following immunological manipulations that eliminate the Th2 component and enhance the type 1 response.

This meta-analysis has numerous advantages in the detection of TB-related IL-4 levels. First, the overall results lend credence to the potential use of IL-4 as a biomarker that may help clinicians objectively assess the severity and active nature of the disease. This will also allow for a clearer understanding of the pathophysiological mechanisms underlying TB. Thus, IL-4 may also be a promising therapeutic target for tuberculosis. Second, this was the largest meta-analysis of the relevant literature, and a subgroup analysis was conducted to provide more reliable results. Data concerning TB in China is important, owing to its increased TB burden; therefore, publications that dealt with Chinese subjects were involved in this process. Third, all the included articles were of medium and high quality, making the evaluation of this study more feasible. Fourth, there was no obvious indication of publication bias, establishing the reliability of the comprehensive results.

However, this study had several limitations. First, the main weakness of the study was that we were unable to address the significant heterogeneity across the investigation of blood IL-4 levels in patients with TB versus healthy controls. Further studies are required to examine unmeasured factors (for example, environment, occupation, smoking status, lifestyle), which may have caused this variability. Second, the study was limited in that any within-the-group comparisons and meta-analyses were unable to be carried out concerning subgroups of TB such as pulmonary versus extrapulmonary, cavitation versus non-cavitation, smear-positive versus smear-negative, and HIV-infected TB versus HIV-uninfected; therefore, it was not possible to examine the link of the disease outcome and the IL-4 profile due to the available data containing fewer observations. Third, the small sample sizes of some subgroups (for example extrapulmonary tuberculosis group and plasma of the IL-4 group) may have led to reduced statistical potency. Owing to data limitations, dose-response relationships between IL-4 concentration and TB risk were not obtained. Fourth, we could not establish a causal link between IL-4 levels and disease severity due to the lack of effective longitudinal cohort studies.

## 5. Conclusion

Serum IL-4 levels were higher in patients with TB than in healthy controls, although there was no significant difference between the levels of IL-4 in the plasma of patients with TB and healthy controls. IL-4 concentration was higher in patients with active TB than those with latent TB. Finally, more detailed experiments are required to determine the association between IL-4 levels and TB risk.

## Acknowledgments

We thank Bullet Edits Limited for the linguistic editing and proofreading of the manuscript.

## Author contributions

**Conceptualization:** Jie He.

Data curation: Jie He.

Formal analysis: Jie He.

Funding acquisition: Jie He.

Investigation: Jie He.

Methodology: Jie He, Lingmeng Song.

Project administration: Jie He.

Resources: Jie He.

Software: Jie He, Lingmeng Song, Pengcheng Zheng.

Supervision: Jie He.

Validation: Jie He, Pengcheng Zheng.

Visualization: Jie He.

Writing – original draft: Jie He, Pengcheng Zheng.

Writing – review & editing: Jie He, Lingmeng Song.

## Supplementary Material
