# Supplementary material for: Interleukin-4 expression is increased in patients with tuberculosis: A systematic review and meta-analysis
Source: Medicine (Baltimore). 2023 Jun 16;102(24):e34041. doi: 10.1097/MD.0000000000034041 (PMC10270521; doi:10.1097/MD.0000000000034041)
Supplement: Supplementary file 1 [file medi-102-e34041-s001.pdf]

Supplementary Table 1.   Search strategies in each database

Database	Search strategies	No. of studies
**PubMed**	#1 "Tuberculosis"[Mesh] OR tuberculosis [Title/Abstract] OR TB[Title/Abstract]<br><br>#2 "Interleukin-4"[Mesh] OR Interleukin-4[Title/Abstract] OR IL-4[Title/Abstract]<br>#3 #1AND #2	955
**EMBASE**	#1 'tuberculosis'/exp OR tuberculosis: ti, ab, kw OR tb: ti, ab, kw<br><br>#2 'interleukin 4'/exp OR 'interleukin 4':ti,ab,kw OR 'il 4':ti,ab,kw<br><br>#3 #1AND #2	950
**VIP**	(M=(tuberculosis or TB) OR R=(tuberculosis or TB)) AND (M=(Interleukin-4 or IL-4) OR R=(Interleukin-4 or IL-4))	24
**Web of Science**	#1 ((TS=(tuberculosis)) OR TI=(tuberculosis or TB)) OR AB=(tuberculosis or TB)<br><br>#2 ((TS=(Interleukin-4)) OR TI=(Interleukin-4 or IL-4)) OR AB=(Interleukin-4 or IL-4)<br><br>#3 #1 AND #2	1098
**CNKI**	(SU=tuberculosis OR TKA=tuberculosis or TB) AND (SU=Interleukin-4 OR TKA=Interleukin-4 or IL-4)	218
**Wan Fang**	(Subject :(tuberculosis) or title or keyword :(tuberculosis or TB)) and (Subject :(Interleukin-4) or title or keyword :(Interleukin-4 or IL-4))	64
